# Palpitations and Asthenia Associated with Venlafaxine in a CYP2D6 Poor Metabolizer and CYP2C19 Intermediate Metabolizer

**DOI:** 10.1155/2017/6236714

**Published:** 2017-10-16

**Authors:** Sofia Garcia, Michael Schuh, Anvir Cheema, Herjot Atwal, Paldeep S. Atwal

**Affiliations:** ^1^College of Pharmacy, University of Florida, Gainesville, FL, USA; ^2^School of Health Sciences, College of Medicine, Mayo Clinic, Jacksonville, FL, USA; ^3^Department of Clinical Genomics, Center for Individualized Medicine, Mayo Clinic, Jacksonville, FL, USA

## Abstract

Cardiotoxicity has been extensively reported in venlafaxine (VEN) overdoses. Asthenia is also among the common side effects described for this antidepressant. VEN is metabolized mainly by CYP2D6 and to a minor extent by CYP2C19 to the major active metabolite O-desmethylvenlafaxine (ODV). Altered pharmacokinetic parameters in patients with polymorphisms in the* CYP2D6* and* CYP2C19* genes that result in decreased enzymatic activity have been documented. Here we describe a patient case of VEN associated palpitations and asthenia. The patient takes VEN extended release 150 mg twice daily. Genotyping confirmed the patient is a poor metabolizer for CYP2D6 and an intermediate metabolizer for CYP2C19. We propose that the palpitations and asthenia are related to sustained VEN exposure due to reduced metabolism.

## 1. Introduction

VEN is a serotonin-norepinephrine reuptake inhibitor (SNRI) prescribed to treat depression [1]. VEN is also used off label for the treatment of posttraumatic stress disorder (PTSD) [2, 3]. Among adverse events reported for this medication, asthenia appears to occur in at least 10% of the patients [4]. Cases of VEN toxicity are associated with QTc prolongation and other cardiovascular presentations [5, 6]. VEN is mainly eliminated via the CYP enzyme system with CYP2D6 and CYP2C19 being the main enzymes involved [7]. Patients with impaired VEN metabolism especially CYP2D6 reduced activity can result in supratherapeutic serum levels of VEN, placing the patient at high risk for adverse effects [8–10]. Furthermore, fatal poisoning due to significant reduction of metabolic capacity in CYP2D6 and CYP2C19 enzymes has also been documented in the literature [11]. The deceased (a 34-year-old Caucasian male) was taking venlafaxine XR 150 mg twice daily. Genotyping revealed the subject has* CYP2D6* (^*∗*^4/^*∗*^5; 1 gene copy) and* CYP2C19* (^*∗*^2/^*∗*^2) which corresponded to poor metabolizer phenotype for both enzymes. Serum level of VEN was extremely elevated and the concentrations of its metabolites were altered as a result of the reduced activity of CYP2D6 and CYP2C19. It seems that the deceased had elevated VEN blood levels for a prolonged period of time before cardiac arrest happened. The authors concluded that the unintended death of the subject could be attributed to the impaired metabolism of VEN [11]. In the light of these reports, we present a patient case of severe asthenia and palpitations associated with the use of VEN in a CYP2D6 poor metabolizer and CYP2C19 intermediate metabolizer.

## 2. Case Presentation

The patient is a 58-year-old Caucasian male with a history of depression and complex posttraumatic stress disorder (PTSD) that presented for a medical therapy management (MTM) consult. The patient expressed feeling intermittent palpitations associated with exertional dyspnea and added that he had been experiencing asthenia and lack of endurance since early childhood; however his fatigue symptoms worsened when he started VEN around twelve years ago. The patient was treated for bipolar disorder, which he believes was misdiagnosed. Additionally, he disclosed that many of the factors contributing to his PTSD in the past are no longer present and for these reasons he has been weaned off from some of the medications prescribed for bipolar disorder and PTSD. Patient's current medications are as follows: venlafaxine XL 150 mg twice daily, adalimumab 40 mg every 15 days, and lamotrigine 125 mg daily. He described his weakness as being unable to do any type of physical activity for a prolong period of time and he experiences pain in his calves by just standing. Contributing to his fatigue, the patient has a history of ankylosing spondylitis. The patient was recently started on coenzyme Q10 400 mg daily and L-carnitine 300 mg daily by the referring physician to aid with his fatigue prior to the MTM consult. He has no history of diabetes, dyslipidemia, or hypertension.

## 3. Medical Evaluation

On clinical examination, the palpitations were not associated with QTc prolongation (QTc = 443 msec). Electrocardiogram (holter) examination revealed symptoms of skipped beats and heart racing which were associated with sinus rhythm and occasional premature atrial beats. He was in sinus tachycardia at 101 beats per minute with normal intervals and normal axis. There were no other abnormalities. He has no associated syncope or presyncope. He also underwent a dobutamine stress test. His ejection fraction response was from 60% at rest to 80% at peak stress. Peak heart rate was 150 bpm, which was 93% of the maximum predicted heart rate. His hemoglobin A1C was borderline elevated at 5.5%; blood glucose was 111 mg/dL (normal). Blood urea nitrogen, creatinine, and urinary profile were all within normal limits. Antinuclear antibodies and other immunology blood testing were unremarkable. Patient's alpha-tocopherol was elevated at 24.9 mg/L. Also serum calcium level was slightly elevated at 10.3 mg/dL. Vitamin D levels were not available; however a pelvic X-ray suggested normal bone mineral density.

## 4. Pharmacogenomic Analysis

Genomic testing was performed from a buccal swab sample using a commercial laboratory. Briefly, genomic DNA was isolated from a buccal swab and the relevant genomic regions were amplified by polymerase chain reaction. Analysis of* CYP1A2, CYP2B6, CYP2C19, CYP3A4,* and* OPRM1* was compiled using a custom xTAG assay (Luminex Molecular Diagnostics). Analysis of* CYP2D6* was completed using xTAG kits. The following genetic variants can be observed using this assay:* CYP1A2:* −3860G>A, −2467>delT, −739T>G, −729C>T, −163C>A, 125C>G, 558C>A, 2116G>A, 2473G>A, 2499A>T, 3497G>A, 3533G>A, 5090C>T, 5166G>A, and 5347C>T;* CYPB6: *^*∗*^1, ^*∗*^4, ^*∗*^6, and ^*∗*^9;* CYP2C19: *^*∗*^1, ^*∗*^2, ^*∗*^3, ^*∗*^4, ^*∗*^5, ^*∗*^6, ^*∗*^7, ^*∗*^8, and ^*∗*^17;* CYP2C9: *^*∗*^1, ^*∗*^2, ^*∗*^3, ^*∗*^4, ^*∗*^5, and ^*∗*^6;* CYP2D6: *^*∗*^1, ^*∗*^2, ^*∗*^2A, ^*∗*^3, ^*∗*^4, ^*∗*^5, ^*∗*^6, ^*∗*^7, ^*∗*^8, ^*∗*^9, ^*∗*^10, ^*∗*^11, ^*∗*^12, ^*∗*^14, ^*∗*^15, ^*∗*^17, and ^*∗*^41, gene duplication;* CYP3A4: *^*∗*^1, ^*∗*^13, ^*∗*^15A, and ^*∗*^22;* OPRM1* 118A>G. The patient carried* CYP2D6 *^*∗*^4/^*∗*^9 and* CY2C19 *^*∗*^1/^*∗*^2 consistent with poor CYP2D6 metabolizer and intermediate CYP2C19 metabolizer phenotypes.

## 5. Antidepressant and Pharmacogenomics Driven Recommendations

Patient's genetic test results established a phenotype of CYP2D6 poor metabolizer and CYP2C19 intermediate metabolizer. Due to reports of cardiotoxicity in VEN overdose and in poor metabolizer patients [12, 13], our recommendations included switching the patient to alternative agents. Antidepressants with alternative metabolic pathways were considered. One of the recommended options was desvenlafaxine, a synthetic form of ODV which is a US Food and Drug Administration (FDA) approved medication in the treatment of depression [14, 15]. Desvenlafaxine was recommended since metabolism does not involve catalysis by CYP2D6 [16]. Other alternatives considered were sertraline or citalopram based on recommendations published by Swen et al. [17]. Additional suggestions were given to gradually tapper down the patient from VEN based on recommendations published by Shelton [18].

## 6. Discussion

It has been suggested that cardiotoxicity can be expected in patients on VEN and with poor metabolism in the CYP2D6 enzyme [12, 13]. VEN is metabolized to O-desmethylvenlafaxine (ODV) mainly by CYP2D6 [19, 20], but it can also be metabolized by CYP2C19 [20]. ODV is an active metabolite with similar pharmacological activity as the parent drug VEN and a half-life of about 10 hours [15, 21]. The target therapeutic concentration in serum for the sum of VEN + ODV ranged between 195 and 400 *μ*g/L [8]; however lower concentration ranges have also been suggested (125 and 400 *μ*g/L) [22]. ODV undergoes further demethylation to N,O-didesmethylvenlafaxine (NODV) mediated via CYP3A4 and CYP2C19. Additionally, VEN can undergo N-demethylation to form N-desmethylvenlafaxine (NDV) catalyzed by CYP3A4 and CYP2C19 [7]. NDV, a minor metabolite, has been associated with weak norepinephrine and serotonin reuptake inhibition [21]. VEN elimination is mainly through urinary excretion (85%) [23]. Elimination entails biotransformation to ODV. Unconjugated and conjugated ODV (29% and 26%, resp.) are the major metabolites excreted in urine. However, 27% of other minor inactive metabolites are also found [24]. NDV is found in about 1% and only 4.7% of unchanged VEN appear in human urine [21, 23]. CYP2D6 poor metabolizers (PMs) have shown a significant decrease in oral clearance compared to extensive metabolizers (EMs). CYP2D6 PMs exhibit higher VEN, lower ODV, higher NDV plasma concentrations, and lower ratio of ODV/VEN compared to EMs [8–10, 12, 25]. Our patient was consistent with poor and intermediate metabolizer of CYP2D6 and CYP2C19, respectively. Even though serum levels of VEN, ODV, or NDV in the patient were not obtained, high levels of VEN, low levels of ODV, and high levels of NDV are expected in this patient based on previous reports [8–10]. The patient is taking a high dose of the extended release formulation. The recommended maximal dose for depression is 225 mg/day but doses up to 300 mg/day have been administered for the off-label use of PTSD [2, 3]. We posit that the impaired metabolism of VEN and the high dose of medication that the patient is receiving can place the patient at increased risk of experiencing side effects. Multiple reports of cardiotoxicity have been documented in the literature with the use of VEN (palpitations, shortness of breath, proarrhythmias, and QT interval prolongation) [5, 12, 26]. In a study comparing VEN overdose to overdoses caused by other antidepressants, VEN was significantly associated with higher heart rate (*P* < 0.0001) [27]. Our patient presented mild tachycardia upon evaluation (HR = 101 bpm). Also of concern, the patient reported a previous history of QT interval prolongation (resolved). Patient QTc was 443 msec which is close to the accepted upper limit of 450 msec in adult males [28]. A similar pattern was observed in a previous electrocardiogram (holter) performed over 12 years ago in this patient. It is not clear from patient's record whether the patient was taking VEN at that time. The possibility that adalimumab might have a role in the mild tachycardia the patient is experiencing cannot be discarded. Palpitation has been also reported in less than 5% of the patients that take adalimumab [29]. However, we propose that the elevated heart rate is associated with high levels of VEN due to the impaired metabolism. No significant drug-drug interactions were found with patient's concomitant medications. Neither lamotrigine nor adalimumab are metabolized by the CYP450 enzymes. Lamotrigine is mainly metabolized via glucuronic acid conjugation [30] and adalimumab seems to be removed by opsonization via the reticuloendothelial system [31]. Therefore additional impact on VEN metabolism due to drug interactions is not anticipated.

The patient also is experiencing severe asthenia. Even though our patient has a significant past medical history of weakness and fatigue, the patient reported worsening of his symptoms after VEN initiation. Several reports of asthenia in patients on VEN have been published [4, 32–35]. Asthenia has also been reported in low frequency in patients taking lamotrigine [30]. However, it is not clear whether it is contributing to patient's symptoms. It is more likely that the patient is experiencing worsening of asthenia and cardiovascular symptoms as a result of accumulation of VEN. The accumulation is related to impaired metabolism by CYP2D6 and CYP2C19 to some extent and the high dose of VEN. Favoring our hypothesis, a fatal drug poisoning case in a patient that was poor metabolizer for both CYP2D6 and CYP2C19 was published by Jornil et al. [11]. The authors attributed the unintended death of the subject to VEN high concentration as a result of the reduced metabolic capacity. There are significant similarities of this case to our patient that are worth highlighting: VEN high dose (150 mg twice daily) of extended release formulation and impaired activity of CYP2D6. Even though our patient was not a CYP2C19 PM, it is arguable that our patient might have some reduction in the activity of the CYP2C19 enzyme. Based on this forensic case and previous reports in CYP2D6 poor metabolizers, it is tempting to state that our patient is experiencing severe asthenia and palpitations as a result of high plasma levels of VEN due to impaired metabolism. It would be necessary to obtain serum levels for VEN, ODV and NDV to confirm. Unfortunately, these levels were not available and this remains a limitation of this study.

In summary, we present a case of severe asthenia and palpitations. VEN appears to be associated with these side effects in a CYP2D6 poor and CYP2C19 intermediate metabolizer. Several studies mentioned in this report have suggested cardiotoxicity associated with VEN overdose and in CYP2D6 poor metabolizers. This report supports the clinical utility of determining a patient's genotype in order to optimize patient therapy by choosing optimal agents and preventing side effects or toxicity. Finally, the involvement of a pharmacist with expertise in pharmacogenomics can be helpful in guiding adjustments based on these increasingly recognized “gene-drug” interactions.
